# High levels of anti-*Leishmania* IgG3 and low CD4^+^ T cells count were associated with relapses in visceral leishmaniasis

**DOI:** 10.1186/s12879-021-06051-5

**Published:** 2021-04-20

**Authors:** Renata Caetano Kuschnir, Leonardo Soares Pereira, Maria Rita Teixeira Dutra, Ludmila de Paula, Maria Luciana Silva-Freitas, Gabriela Corrêa-Castro, Simone da Costa Cruz Silva, Glaucia Cota, Joanna Reis Santos-Oliveira, Alda Maria Da-Cruz

**Affiliations:** 1 Laboratório Interdisciplinar de Pesquisas Médicas, Instituto Oswaldo Cruz, FIOCRUZ, Rio de Janeiro, Rio de Janeiro Brazil; 2 Hospital Eduardo de Menezes, Fundação Hospitalar do Estado de Minas Gerais, Belo Horizonte, Minas Gerais Brazil; 3 Núcleo de Ciências Biomédicas Aplicadas, Instituto Federal de Educação, Ciência e Tecnologia – IFRJ, Rio de Janeiro, Rio de Janeiro Brazil; 4 Instituto Nacional de Infectologia Evandro Chagas, FIOCRUZ, Rio de Janeiro, Rio de Janeiro Brazil; 5 Instituto René Rachou, FIOCRUZ, Belo Horizonte, Minas Gerais Brazil; 6 Disciplina de Parasitologia, DMIP, Faculdade de Ciências Médicas, UERJ, Rio de Janeiro, Brazil; 7 Rede de Pesquisas em Saúde do Estado do Rio de Janeiro/ FAPERJ, Rio de Janeiro, Brazil

**Keywords:** Visceral leishmaniasis, Relapses, Clinical follow-up, Immune response

## Abstract

**Background:**

Visceral leishmaniasis (VL) is severe and potentially fatal. Brazil is one of the countries with the greatest endemicity for the disease in the world. The reduction of CD4^+^ T lymphocytes, B cells activation and high levels of inflammatory cytokines (IL-6/IL-8/TNF/IL-1β), plasma LPS, soluble CD14, anti-*Leishmania* IgG3 and low leptin levels are involved in the immunopathogenesis of VL, most associated with severe VL. Despite relapses occurring in about 4–5% of patients with VL not associated with HIV infection, the factors underlying relapses are little known. Our aim was to identify clinical, laboratory and immunological parameters that may be associated with recurrences in VL.

**Methods:**

Fifteen VL patients recruited from Hospital Eduardo de Menezes (BH-MG) were grouped into relapsing (R-VL, *n* = 5) and non-relapsing (NR-VL, *n* = 10) and evaluated during active disease, immediately after treatment (post-treatment) and 6 months post-treatment (6mpt). Clinical and laboratory data obtained from medical records were correlated with CD4^+^ and CD8^+^ T cell counts and anti-*Leishmania* Igs and IL-6 plasma levels and compared to those parameters of ten healthy controls.

**Results:**

During the active phase of VL, despite similarity in the clinical symptoms, the rates of thrombocytopenia, elevated transaminases (AST and ALT) and hyperbilirubinemia were higher in the NR-VL group compared to R-VL (*p* < 0.05), a profile reversed during the post-treatment phase. All patients had low CD4^+^ T counts in active phase, however, NR-VL patients had a higher gain of this cell type than R-VL in the post-treatment (*p* < 0.05). There was a significant reduction in IgG3 levels during the follow-up in the NR-VL group compared to the R-VL, especially at 6mpt (*p* < 0.05). In addition, IgG3 levels were negatively correlated with CD4^+^ T counts in the R-VL group (*r* = − 0.52). Elevated levels of IL-6 were observed in active VL and correlated with clinical markers of severity.

**Conclusions:**

During active phase of VL, the NR-VL patients presented more severe laboratorial abnormalities compared to R-VL, probably because the latter had already received previous treatment. On the other hand, R-VL exhibited greater impairment of immune reconstitution and a high degree of B lymphocyte activation, which must be a factor that favored relapses.

**Supplementary Information:**

The online version contains supplementary material available at 10.1186/s12879-021-06051-5.

## Background

Visceral leishmaniasis (VL) is caused by *Leishmania* (*L*.) *infantum* in Brazil, being transmitted to mammals by *Lutzomyia longipalpis* sand fly [1]. VL is endemic in more than 90 countries or territories, however, in 2017, few countries as Brazil, Ethiopia, India, Kenya, South Sudan and Sudan have concentrated more than 90% of the cases [2]. In the Americas, 3562 new cases of VL were diagnosed in 2018 and Brazil was responsible for 97% of them [3]. Belo Horizonte, located in Minas Gerais state, is one of the Brazilian cities with the highest number of VL patients, with 2378 reported cases from 2007 to 2019 [4].

Pathogen and host’s immune system interaction leads to different clinical presentation, predisposing a high variety of outcomes, since asymptomatic disease to high severity and risk of death. Commonly, most patients affected by VL respond well to anti-*Leishmania* treatment and evolve to the remission. However, there are those who evolve to the severe form of VL, with a high lethality rate [5]. In addition, relapse is also observed in medical practice [6, 7].

Clinical and laboratory markers such as age, bleeding, edema, jaundice, dyspnea, bacterial infection, HIV/AIDS co-infection, leukocyte count below 1500 cells/mm^3^, thrombocytopenia below 50,000 cells/mm^3^ and renal failure have also been linked to VL severity [8, 9]. Also, several soluble molecules, such as elevated levels of IL-2R, IL-1β, IL-6, IL-8, IL-27, TNF, soluble CD14 (sCD14) [10–13], soluble CD163 (sCD163) [14] and specific anti-*Leishmania* immunoglobulins [15, 16], as well as low leptin levels [17] have already been associated with severity of VL. Interestingly, IL-6 levels were also associated with risk of death in VL [12].

Indeed, besides parasite specific immunosuppression, the exuberant inflammatory condition constitutes the key mechanism in the physiopathology of *L. infantum* infection. Similar to what is seen in sepsis, severe dengue and severe malaria, high degree of cell activation and high levels of cytokines are seen in the active VL [12, 18–20]. These mechanisms contribute to the immune response impairment, which in turn have a negative impact on the effector capacity to control the parasite. Therefore, these features can influence the clinical evolution of VL patients, in terms of clinical cure status, severity/death and disease recurrences/relapses.

Relapses are characterized by the resurgence of signs and symptoms after an initial improvement of a disease manifestation [21]. It is considered a risk factor for death in VL, especially in HIV-co-infected patients [22, 23]. Recently, our group demonstrated that the maintenance of high levels of cell activation, microbial translocation products, anti-*Leishmania* IgG3 and a low CD4^+^ T cell reconstitution could be associated with VL relapse in VL/HIV coinfected patients [23]. Moreover, this low immune reconstitution has been related to a greater impairment of the thymic output among VL/HIV-relapsing patients [24]. The inability to reconstitute the effector response seems to exert a key role in the VL relapses.

In Brazil, the official frequency of relapses after VL treatment among non HIV-infected patients is underestimated, although the compulsory notification form has a specific field to inform this clinical presentation [25]. In the literature, the vast majority of studies are related to therapeutic failure reports [26]. There are few VL relapses reports and those which have it, present a great variability in the incidence of relapses according to the population and regions studied [27–30]. In a Spanish cohort, VL relapse rate was 12% [27] while in Sudan, the clinical follow-up during an epidemic in Babar showed 5.7% of relapses, most of patients concentrated in few families [31]. A previous Brazilian study showed relapse in 2.3% of children diagnosed with VL between 2006 and 2011 [30]. Based on secondary data, an analysis of VL cases also in Brazil from 2001 to 2010 showed that relapses occurred in 3.1% of patients not co-infected with HIV [29].

Factors underlying clinical relapses in VL alone have not been deeply addressed. Clinical characteristics as male gender, extreme of age and discreet reduction of splenomegaly were risk factors associated with relapses in Indian VL patients [32, 33]. In Georgia, a pediatric cohort showed that children younger than one-year-old had a higher chance of relapse as the ones who were late diagnosed [34]. More recently, a Brazilian study showed that in addition to HIV infection, thrombocytopenia, lower limb edema and secondary pneumonia were factors independently associated with relapse [28].

VL relapsing patients should not achieve an effector immune response able to maintain the remission of the disease. Herein, we actively monitored patients with VL, followed from the active phase of the disease  up to 12 months after treatment and confirmed the presence of two distinct groups: those with primary VL without relapse along 6 months (assumed as single/life episode) and those with two or more VL episodes/life, either with relapse identified at the cohort enrollment or during the follow-up period. Our aim was to identify possible differences in clinical and immunological parameters that could infer mechanisms involved in recurrence of VL. We evidenced that after therapy, both groups improved hematological and biochemical parameters and reduced IL-6 levels. In contrast, VL relapsing patients maintained increased IgG3 levels besides lower CD4^+^ T cell counts in comparison to non-relapsing VL patients. These results suggest that VL relapsing patients keep a B cellular activation status along with a deficient T cell compartment reconstitution.

## Methods

### Casuistic and study design

Fifteen VL patients were recruited for a prospective cohort study carried out in an infectious diseases referral hospital in Belo Horizonte, MG, Brazil (Hospital Eduardo de Menezes – Fundação Hospitalar do Estado de Minas Gerais/ HEM-FHEMIG) from May 2018 to September 2019 (Flow diagram). The inclusion criteria in this cohort was presence of fever, cytopenia or splenomegaly, age over 18 years, independently of gender. Pregnant women and patients with HIV-infection were excluded. The leishmaniasis diagnosis was confirmed by direct visualization of amastigotes in bone marrow aspirate or presence of anti-*Leishmania* antibodies in serum. The patients were divided in two groups: those who had experienced only one VL episode throughout life (non-relapsing – NR) and those experiencing more than one VL episode, either previously or during the prospective follow-up (relapsing – R – group). Also, ten healthy subjects were included (HS, *n* = 10) as controls.

Cure was defined clinically by the disappearance of fever associated to recovery of cytopenia and splenomegaly involution, if present. Relapses were parasitologicaly confirmed by direct examination or culture in NNN medium in patients presenting resurgence of fever, worsening cytopenia or increased splenomegaly, compared to the previous condition, as defined by Cota et al. [21]. Patients with clinical symptoms and parasitological confirmation of active VL were included only after appropriate written informed consent was obtained. This study was approved by the Ethical Committees of Instituto Oswaldo Cruz - FIOCRUZ, HEM-FHEMIG and Instituto René Rachou – FIOCRUZ.

Patients were followed prospectively for 6 months and evaluated in three visits: active phase of the disease (before anti-*Leishmania* treatment), after anti-*Leishmania* treatment, and 6 months after treatment (6mpt). Some participants were followed up to 12 months after treatment (NR-VL = 5 and R-VL = 3). The post-treatment time point varied from patient to patient, due to differences in therapeutic regimens, but for all of them, this moment refers to the last day of medication (Table 1).

**Table 1:** Clinical characteristics of non-relapsing and relapsing VL patients

	Patient	Comorbidities	Active phase treatment	Cumulative Amph B dose in VL active phase	Previous episodes of VL	Time between first VL episode and current active phase	Relapse during follow-up	Time between current active phase and relapse	Total number of VL episodes	Total follow-up
**Non-Relapsing**	**VL01**	Smoker Previous marijuana use Occasional use of alcohol	Amph. B deoxychol 50 mg/day per 12 days followed by Liposomal Amph. B 20 mg/kg	28.5 mg/kg	No	NA	No	NA	1	12 months
	**VL02**	No information	Liposomal Amph. B 20 mg/kg	20 mg/kg	No	NA	No	NA	1	12 months
	**VL03**	Occasional use of alcohol Positive serology for Chagas Disease	Amph. B deoxychol 50 mg/day per 3 days followed by Liposomal Amph. B 20 mg/kg	21.7 mg/kg	No	NA	No	NA	1	12 months
	**VL04**	Virchowian hanseniasis ADHD Development delay – infantilized behavior	Amph. B deoxychol 50 mg/day per 4 days followed by Liposomal Amph. B 20 mg/kg	22 mg/kg	No	NA	No	NA	1	12 months
	**VL05**	Dyslipidemia Past of drug addiction	Amph. B deoxychol - 1000 mg^a^		No	NA	No	NA	1	Post-treatment
	**VL08**	Arterial hypertension Smoker Alcoholic	Amph. B Lipid Complex 20 mg/kg	20 mg/kg	No	NA	No	NA	1	12 months
	**VL11**	Alcoholic Ex-smoker Past of schistosomiasis and gonorrhea	Amph. B Lipid Complex 200 mg/day for 4 days followed by Liposomal Amph. B 20 mg/kg (in 7 weeks)	20 mg/kg	No	NA	No	NA	1	6 months
	**VL12**	Drug addicted	Amph. B deoxychol 150 mg followed by Liposomal Amph. B 20 mg/kg	23 mg/kg	No	NA	No	NA	1	6 months
	**VL13**	Alcoholic Smoker	Meglumine antimoniate 20 mgSb+ 5/Kg/day for 3 days followed by Liposomal Amph. B 20 mg/kg	20 mg/kg	No	NA	No	NA	1	Post-treatment
	**VL14**	Arterial hypertension Ex-smoker	Liposomal Amph. B 20 mg/kg in7 days	20 mg/kg	No	NA	No	NA	1	Post-treatment
**Relapsing**	**VL06**	Arterial hypertension Diabetes mellitus type 2 Metabolic syndrome	Amph. B Lipid Complex 20 mg/kg	20 mg/kg	Yes (1)	4 months	No	NA	2	12 months
	**VL07**	Occasional use of alcohol	Amph. B Lipid Complex 40 mg/kg	40 mg/kg	Yes (3)	15 months	Yes Jan, 2019	4 months	5	12 months
	**VL09**	Chronic renal disease Hypersplenim Past of schistosomiasis	Amph. B Lipid Complex 20 mg/kg	20 mg/kg	Yes (2)	15 months	Yes Oct, 2019	12 months	4	12 months
	**VL10**	Chagasic cardiac insufficiency	Amph. B Lipid Complex 25 mg/kg	25 mg/kg	No	NA	Yes Feb, 2019	4 months	2	6 months
	**VL15**	Ex-smoker Pulmonary hypertension Subclinic hypothyroidism	Liposomal Amph. B 40 mg/kg	40 mg/kg	Yes (2)	5 months	No	NA	3	Post-treatment

*VL* visceral leishmaniasis, *yo* years old, *ADHD* attention deficit hyperactivity disorder, *Amph. B deoxychol* Amphotericin B deoxycholate, *Liposomal Amph. B* Liposomal amphotericin B, *Amph. B Lipid Complex* Amphotericin B lipid complex

^a^no exact dose information available *NA* not applicable

Patient’s data were obtained from the medical records: 1) clinical signs and symptoms - fever, asthenia, hyporexia, weight loss, vomiting, diarrhea, bleeding, pallor, edema, splenomegaly, hepatomegaly, jaundice and underlying bacterial infection; 2) laboratory data - blood cell counts, biochemical tests related to liver and kidney function.

In each study visit, 45 mL of peripheral blood were collected from each patient, divided into 3 tubes with heparin (10 mL each) and 3 tubes with EDTA (5 mL each). The whole blood conserved in EDTA was used for the CD4^+^/CD8^+^ T lymphocytes count, while the plasma in heparin tube was used to evaluate the anti-*Leishmania* IgG immunoglobulins levels and the IgG1 and IgG3 subclasses, as well as the levels of IL-6. The VL therapy was defined on individual basis according to the service routine and Brazilian Minister of Health recommendations [1].

### Absolute T-cell counts quantification

Absolute T lymphocyte counts were determined using the BD Multitest monoclonal antibodies anti-CD45-PerCP, anti-CD3-FITC, anti-CD4-APC, and anti-CD8-PE (BD® Biosciences, Franklin Lakes, NJ, USA) according to the manufacturer’s instructions and as described previously by Silva-Freitas et al. [23]. The counts were acquired using a FACSCalibur and later the FACSVia. The data were analyzed with Multiset software (BD®, USA). The results are expressed as the number of cells per cubic millimeter (cells/mm^3^) and was performed at the reference service of the Instituto Nacional de Infectologia Evandro Chagas, FIOCRUZ.

### Anti-*Leishmania* immunoglobulin assessment

An ELISA was performed as previously described in Silva-Freitas et al. [23], with some modifications. Briefly, *L. (L.) infantum* (MHOM/BR/1974/PP75) soluble promastigote (40 μg/mL) was used to coat a polystyrene flat-bottom microtiter plate (Nunc-Immuno, Roskilde, Denmark). In this assay, plasma samples from VL patients were diluted as follows: 1:10,000 to IgG, 1:2000 to IgG1 and 1:200 to IgG3. Then, diluted peroxidase-conjugated mouse monoclonal anti-human immunoglobulin G (IgG) (1:1000) (Invitrogen, San Francisco, CA, USA) and diluted monoclonal anti-human IgG1 (1:500) and IgG3 (1:400) (Zymed Laboratories Inc., San Francisco, CA, USA) were used. The absorbance was measured with a Benchmark microplate reader (Bio-Rad Laboratories, Hercules, CA, USA) at 492 nm. The results were expressed as an ELISA index (EI), which is based on the division of the average optical density (OD) of the duplicates of the patient samples, by the average OD obtained from the negative controls.

### Quantitation of IL-6 levels in plasma

IL-6 levels were quantified in plasma samples stored at − 70 °C using a commercial kit (IL-6 Quantikine ELISA, R&D Systems, Minneapolis, Maryland, USA), according to the manufacturer’s recommendations. A standard seven-point curve diluted in calibration reagent, as well as plasma samples were quantified in duplicate. The optical density was determined by the Microplate reader Benchmark equipment (Bio-Rad Laboratories, Hercules, CA, USA) at 450 nm. The results were expressed in picograms per milliliter (pg/mL), and the minimum detection limit was 3.13 pg/mL.

### Statistical analysis

Laboratorial parameters were expressed in medians with interquartile ranges shown in square brackets. The gain of CD4^+^ and CD8^+^ T lymphocytes was performed based on the ratio between the number of cells present at a given time and the number of cells present in the active phase of VL. Comparisons between the two groups of VL patients (NR and R) and between each VL group and the control group were performed using the unpaired and non-parametric, Mann Whitney t-test. Wilcoxon tests for paired variables with skewed distributions were also used for comparisons involving the same individual at different times. Spearman’s test was used for correlation analysis. The statistical analyses were performed using SPSS® version 16 (multivariate analysis) and GraphPad Prism software (version 6.0, San Diego, CA, USA). Differences were considered statistically significant when the *p* value was < 0.05.

## Results

### Clinical and laboratory characteristics of non-relapsing and relapsing visceral leishmaniasis patients in active phase and after anti-*Leishmania* treatment

Fifteen VL patients were enrolled in the study (Table 1). During active VL phase, fever, asthenia and weight loss were reported by 14 (93%), 11 (73%) and 12 (80%) patients, respectively. Splenomegaly was identified in all patients based on physical examination and/or imaging tests, and 12 patients had a concomitant liver enlargement. Considering the severity markers defined by the Brazilian Minister of Health, three patients complained of abdominal pain, two patients reported bleeding prior to hospitalization and one presented hematemesis during the clinical evolution. Ten patients received antibiotic therapy for suspected or confirmed concomitant bacterial infection.

Only one patient completed treatment using amphotericin B deoxycholate, whereas most patients required the use of amphotericin B lipid formulations (Table 1). R-VL patients showed higher accumulated dose during the treatment in active phase, although without significant difference between groups (Table 1 and 2).

**Table 2:** Clinical and laboratory differences between relapsing and non-relapsing visceral leishmaniasis patients

Analyzed parameters	Visceral leishmaniasis patients [median (IQR)]		
	NR (***n*** = 10)	R (***n*** = 5)	***p***-value
**Sex (M/F)**	8/2	4/1	NA
**Age (years)**	38 (30.8–46.5)	44 (30–61)	0.49
**Total number of VL episodes (n)**	1	3 (2–5)	NA
**Accumulated dose of Amph. B in the current active VL phase (mg/kg)**	20 (20–22.5)	25 (20–40)	0.24
**C-reactive protein (mg/dL)**	72 (42.3–154.5)	54.5 (39.5–174.5)	0.76
**Platelet (x10**^**3**^**cel/mm**^**3**^**)**	68 (42.8–80.8)	107 (88–216.5)	**0.01**
**Aspartate aminotransferase (U/L)**	69.5 (56.8–195.8)	38 (24–79)	**0.03**
**Total bilirubin (mg/dL)**	0.9 (0.7–1.0)	0.5 (0.5–0.8)	**0.03**

Regarding liver function, transaminases levels (AST and/or ALT) were augmented in the majority of NR-VL (7 out 10), but in only one out 5 of the R-VL group (*p* < 0.05, Table 2). The total bilirubin was at normal levels in both groups; however, the direct bilirubin (DB) levels were more elevated in NR-VL (7 out 10) than in R-VL, in which only one patient presented a slight increase of DB (Table 2 and Supplementary Table 1). A high degree of inflammatory activity can be inferred from the elevation of C-reactive protein (NR-VL = 72 mg/dL [42.3–154.5 mg/dL] and *R* = 54.5 mg/dL [39.5–174.5 mg/dL]), compared to normal parameters (< 10 mg/dL), with no statistical difference between groups of patients (Table 2 and Supplementary Table 1).

**Fig. 1:**
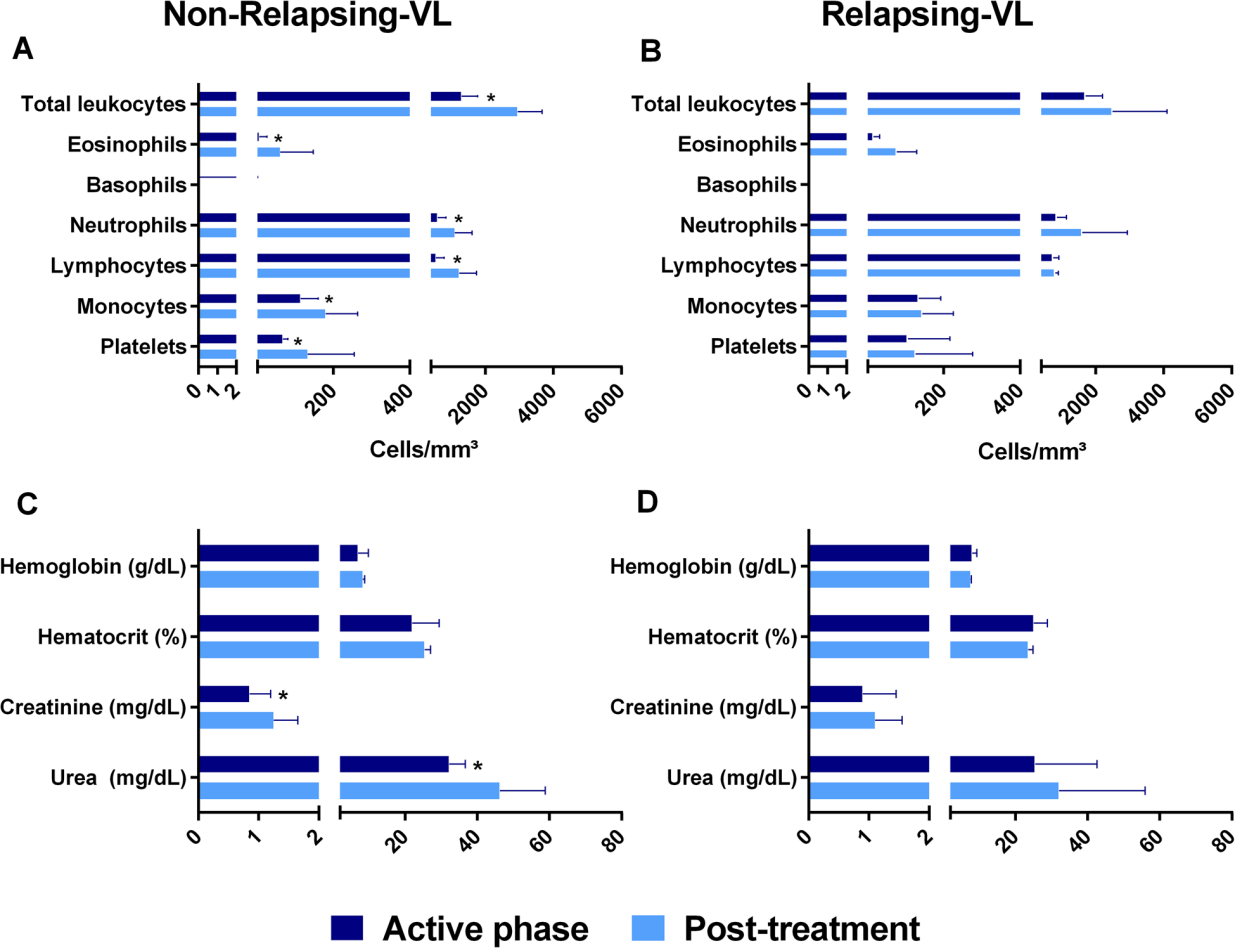
Laboratorial indices of non-relapsing (NR-) and relapsing (R-) visceral leishmaniasis (VL) patients. Comparison between the median values of active phase and early post-treatment of NR (**a** and **c**) and R (**b** and **d**) VL patients. Asterisks denote statistically significant differences between the phases of clinical follow-up: *p < 0.05

Considering hematological parameters, all patients had anemia, leukopenia and thrombocytopenia. NR-VL patients presented the lowest cell types counts compared to R-group, but only differences on platelets counts were statistically significative (*p* < 0.05) (Table 2 and Supplementary Table 1). After treatment, NR-VL patients showed a significant increase in total leukocytes from 1300 cells/mm^3^ [975–1775 cells/mm^3^] to 2950 cells/mm^3^ [1825–3675 cells/mm^3^]. From these, lymphocytes, monocytes, and neutrophils significantly augmented after therapy (*p* < 0.05) (Fig. 1). This increase of leucocytes also occurred in R patients, but the differences were not significant (Fig. 1). Finally, the NR-VL group also showed a significant increase in platelet counts immediately after treatment in relation to the active phase of VL.

### Visceral leishmaniasis relapses were associated with maintenance of low CD4^+^ T-cells

**Fig. 2:**
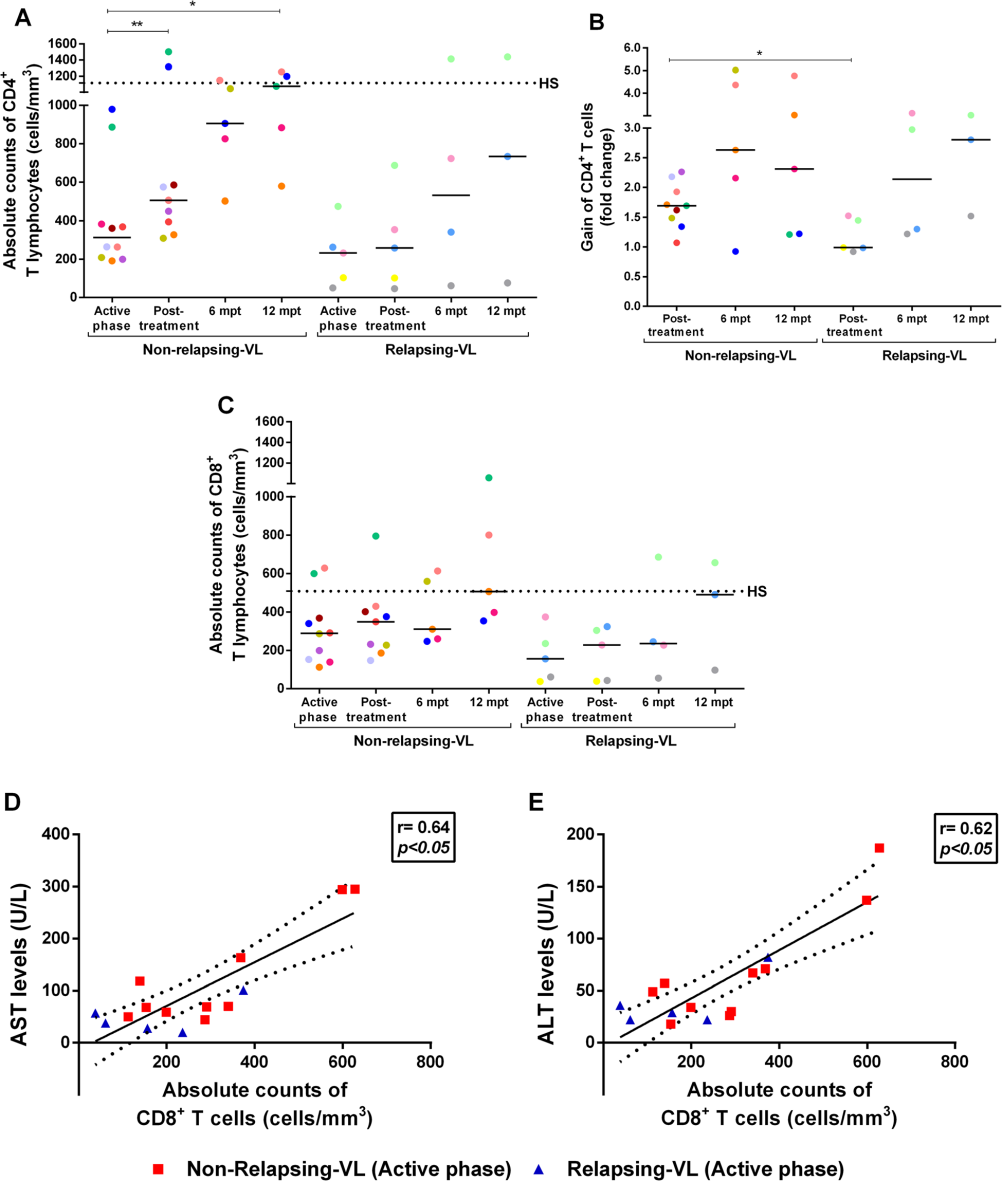
Evaluation of immune impairment in visceral leishmaniasis (VL) patients throughout clinical follow-up. Absolute counts of CD4^+^ (**a**) and CD8^+^ (**c**) T lymphocytes of non-relapsing (NR) and relapsing (R) VL patients**.** Gain of CD4^+^ T lymphocytes during clinical follow-up in relation to the active phase of VL (**b**). Positive correlation between the absolute counts of CD8^+^ T lymphocytes and the aspartate aminotransferase - AST (**d**) and alanine aminotransferase - ALT (**e**) levels during the active phase of VL (Spearman correlation, *r* = 0.64 and *r* = 0.62, respectively, *p* < 0.05). Each point represents a VL patient and each color represents the same patient in the different stages of clinical follow-up. The black dashed line represents the median value of healthy subjects (HS). The horizontal bars represent the median values of each group. Post-treatment: Early post-treatment. Mpt: months post-treatment. Asterisks denote significant differences between the phases of clinical follow-up within the R or NR group itself or even between the R and NR group: *p < 0.05. ** *p* < 0.01

All VL patients, regardless of being from R- or NR-VL group, had lower CD4^+^ T cell counts (NR-VL - 312.5 cells/mm^3^ [205.8–509 cells/mm^3^] and R - 232 cell/mm^3^ [76.5–368.5 cell/mm^3^]) during active VL compared to HS (1115 cell/mm^3^ [630.5–1258 cell/mm^3^]) (Fig. 2a). However, immediately after treatment a significant increase of CD4^*+*^ T-cell counts was observed in the NR-VL group, but not in R group. Likewise, the gain of CD4^+^ T lymphocytes after anti-*Leishmania* treatment was 1.69 times [1.41–2.05 times] in relation to the active phase of the disease (*p* < 0.05); while the gain in patients R was 0.99 [0.95–1.48] (Fig. 2b).

After 6 months of treatment (6mpt), NR-VL patients still maintained higher CD4^+^ T lymphocyte counts than R patients (NR-VL - 906 cells/mm^3^ [664–1097 cells/mm^3^]; R-VL - 532 cells/mm^3^ [131–532 cells/mm^3^]) (Fig. 2a and b). Some patients were followed up to 12mpt and among NR-VL, four patients presented CD4^+^ T counts above than 800 cells/mm^3^ (1074 cell/mm^3^ [731.5–1226 cell/mm^3^] very similar to those found in HS (1115 cell/mm^3^ [630.5–1258 cell/mm^3^]) (Fig. 2a). On the other hand, even at a long term post-therapy, R-VL patients still presented the lowest CD4^+^ T cell counts (734 cell/mm^3^ [76–1438 cell/mm^3^]) (Fig. 2a and b).

Regarding CD8^+^ T lymphocytes, both NR-VL and R-VL patients showed lower numbers of this subpopulation than those found in HS (Fig. 2c) up to 6mpt. At 12mpt, the CD8^+^ T-cells reached counts very similar to those found in HS (Fig. 2c) in both groups.

Interestingly, the CD8^+^ T-cell counts were positively correlated with the transaminase levels in the active phase of the disease: CD8^+^ T-cells and AST (*r* = 0.64; *p* < 0.05) and with ALT (*r* = 0.63; *p* < 0.05 - Fig. 2d and e).

### Relapsing visceral leishmaniasis patients maintain elevated IgG3 anti-*Leishmania* levels

**Fig. 3:**
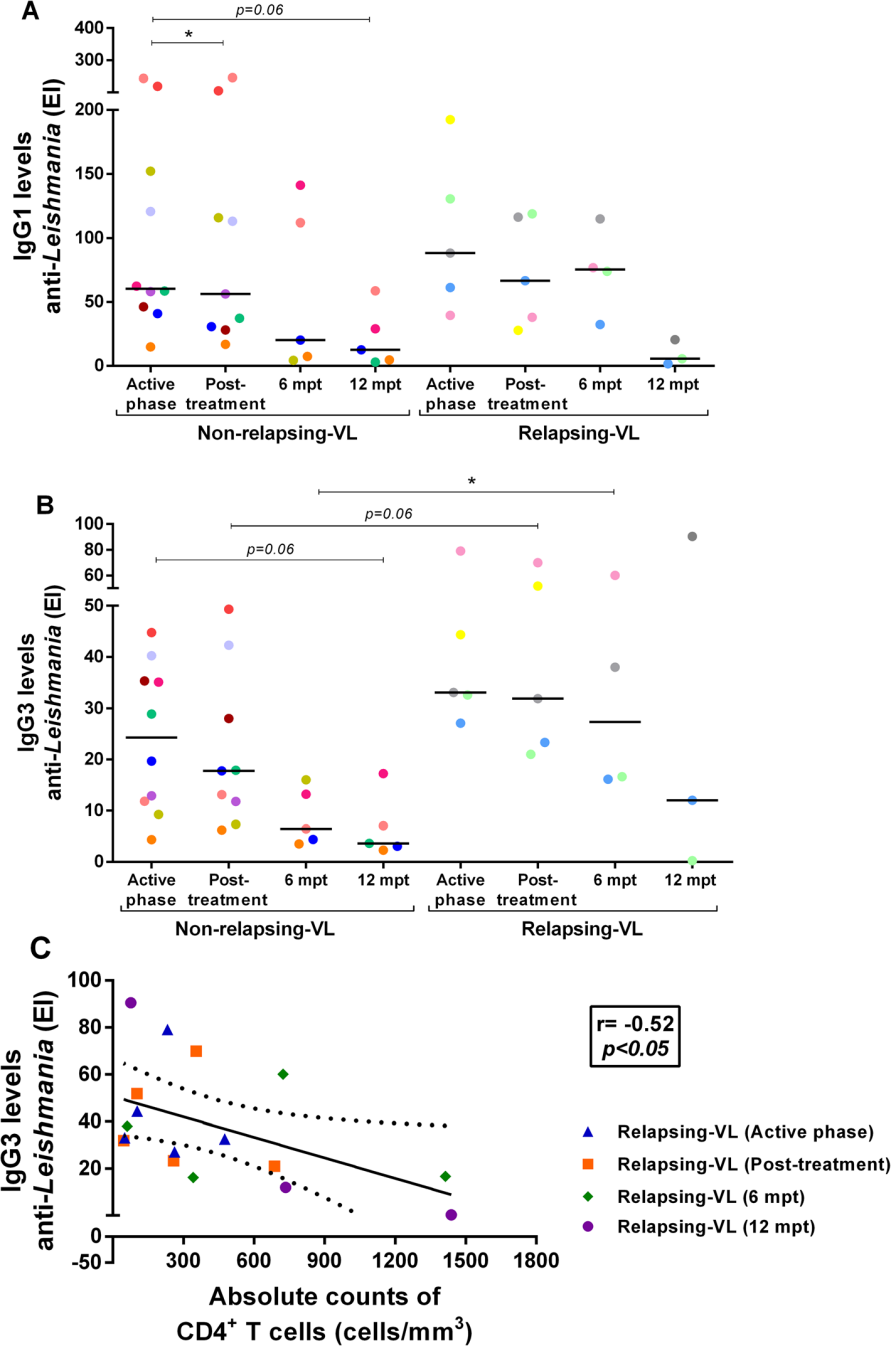
Anti-*Leishmania infantum* Igs levels in non-relapsing (NR) and relapsing (R) VL patients. IgG1 (**a**) and IgG3 (**b**) levels in NR-VL and R-VL patients throughout clinical follow up. Negative correlation between the absolute counts of CD4^+^ T lymphocytes and levels of anti-*L. infantum* IgG3 during all the follow-up (**c**) (Spearman correlation, *p* < 0.05, *r* = − 0.52). Post-treatment: Early post-treatment. Mpt: months post-treatment. Each point represents a VL patient and each color represents the same patient in the different stages of clinical follow-up. The horizontal bars represent the median values of each group. Asterisks denote statistically significant differences between the phases of clinical follow-up within the R or NR group itself or even between the R and NR group: *p < 0.05

The IgG anti-*Leishmania* levels were measured by Elisa Index (EI). Despite no significant difference, NR-VL patients presented lower levels of anti-*Leishmania* IgG1 than R-VL group up to 6mpt (Fig. 3a). It is interesting to note that NR-VL group has already presented a reduction in these levels soon after the anti-*Leishmania* treatment, whereas higher antibodies levels persisted up to 6mpt in R patients (NR-VL = 20.2 [5.8–126.5]; R-VL = 75.4 [42.8–105.3]) (Fig. 3a). After 12 months, IgG1 levels showed a tendency to decrease in the NR-VL group (12.5 [3.8–43.8], *p* = 0.06) in relation to the active phase (Fig. 3a).

In terms of IgG3 levels, NR-VL patients showed a gradual reduction in these levels right after anti-*Leishmania* treatment in relation to the active phase of VL (active phase: 24.3 [11.2–36.6]; post-treatment: 17.8 [9.6–35.1], 6mpt: 6.4 [3.9–14.6], 12mpt: 3.6 [2.7–12.2], *p* = 0.06) (Fig. 3b). However, it is notable that such decrease was higher in NR-VL group when compared to R-VL (*p* < 0.05), whose IgG3 levels remained elevated up to 6mpt (R-VL: active phase: 33.1 [29.9–61.8]; post-treatment: 31.9 [22.2–60.9]; 6mpt: 27.3 [16.3–54.6]). (Fig. 3b).

Finally, IgG3 anti-*Leishmania* levels correlated negatively with CD4^+^ T cell counts (*r* = − 0.52, *p* < 0.05, Fig. 3c) in R-VL patients in all phases of the clinical follow-up, which reinforces that relapsing patients whose CD4^+^ T counts are lower are also those who have higher IgG3 levels.

### IL-6 levels correlated with laboratorial parameters of severity in visceral leishmaniasis patients

**Fig. 4:**
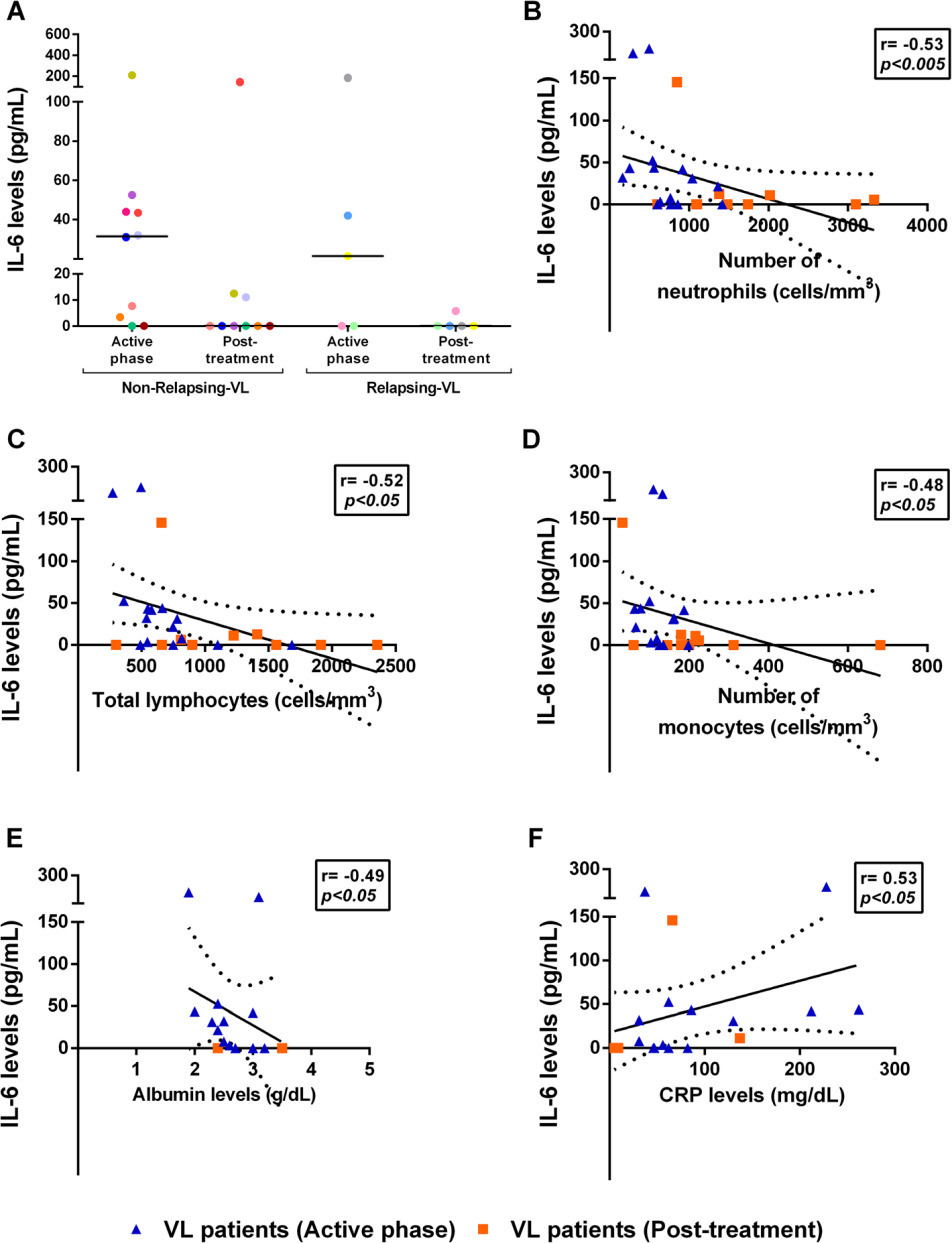
Assessment of IL-6 levels and correlation with laboratory parameters in visceral leishmaniasis (VL) patients. Plasma IL-6 levels in non-relapsing (NR) and relapsing (R) VL patients throughout clinical follow-up (**a**). Negative correlation between the IL-6 levels during VL active phase and early post-treatment and the neutrophil counts (**b,** Spearman correlation, *r* = − 0.53; *p* < 0.005), lymphocyte counts (**c,** *r* = − 0.52, *p* < 0.05), monocyte counts (**d,** *r* = − 0.48, *p* < 0.05 ) and albumin levels (**e,** *r* = − 0.49, *p* < 0.05). Positive correlation between the IL-6 levels at active phase and early post-treatment and C-reactive protein (CRP) levels (**f,** *r* = 0.53, *p* < 0.05). Post-treatment: Early post-treatment. Each point represents a VL patient and each color represents the same patient in the different stages of clinical follow-up. The horizontal bars represent the median values of each group

In the active phase, IL-6 levels were above the minimum detection limit in 8 out 10 NR-VL and in 3 out 5 R-VL in relation to the HS, whose median was 0.1 pg/mL [0.1–165 pg/ml]. However, a reduction in these levels were observed immediately after the treatment in most patients of both groups (Fig. 4a). For both VL groups, IL-6 levels were associated with several VL severity markers, during the active and post-treatment phases. A negative correlation was verified between IL-6 levels and neutrophils (*r* = − 0.53, *p* < 0.05, Fig. 4b), lymphocytes (*r* = − 0.52; *p* < 0.05, Fig. 4c), monocytes (*r* = − 0.48, *p* < 0.05, Fig. 4d) and albumin (*r* = − 0.49; *p* < 0.05, Fig. 4e). On the other hand, IL-6 levels correlated positively with C-reactive protein levels (*r* = 0.53; *p* < 0.05) (Fig. 4f).

## Discussion

There are many studies carried out in India [33], Sudan [32], Georgia [34] and Brazil [28] addressing retrospectively non-HIV infected patients presenting a VL relapsing course, but divergent findings in terms of clinical and laboratorial aspects associated with relapses were found. In the present study, the patients’ symptoms were quite similar in the active phase, and it was not possible to identify significant differences between R- and NR-VL groups. However, more intense laboratorial abnormalities were observed in NR-VL compared to R-VL group. As R-VL patients have previously undergone anti-*Leishmania* treatment, we believe that it may have contributed to a partial clinical recovery, with a reduction in the damage caused by the infection. However, this was not enough to restore the R-VL organic functions.

Splenomegaly at study enrollment was observed in both groups, being a usual manifestation of VL [9]. Splenomegaly and thrombocytopenia have already been identified as markers of VL relapses [28, 32, 33]. NR-VL patients showed significant platelet elevation after treatment, different from observed for R-VL group. It is known that spleen histological disorganization affects its functionality [35] which implies in higher consumption of platelets, as a confounding factor, resulting in elevated parasitemia [9], and immunodepression related to its immunity function [32].

The responsible medical team decided each patient treatment in an individualized way, based on clinic characteristics and side effects of the avaiable drugs. It is important to highlight that although Amph. B Lipid Complex is not considered a drug of first choice in VL treatment in international and Brazilian official guidelines, there are studies that were able to show the effectiveness of this drug in VL cases, including in HIV coinfected patients [36–39]. Therefore, we do not believe that the treatment with this specific Amph. B formulation is related with the occurrence of new VL episodes in these patients.

After VL treatment, a recovery of bone marrow cellularity was observed in NR-VL patients, especially with an increase in leukocytes levels compared to the active phase of VL. A significant and faster gain of CD4^+^ T cells was observed in NR-VL patients immediately after anti-*Leishmania* treatment, but not in R-VL patients. Similar results were observed in VL/HIV coinfection [23]. This deficit in the recovery of CD4^+^ T lymphocytes can be related to an impaired input of cells originated from bone marrow, deficient replication rate or thymic dysfunction [24, 40, 41]. Low CD4^+^ T cell counts in VL/HIV coinfected patients during the active phase of VL was a predictor of a poor prognosis: death or recurrence [42, 43]. Thus, patients who are unable to restore the CD4^+^ T counts may be prone to relapse. Interestingly, in the current series, all VL patients maintained low CD8^+^ T lymphocyte counts up to 6 months of follow-up, in relapsing and not relapsing groups. Whether CD8^+^ T cells contribute to protection, immunopathogenesis or even to the immunosuppression in VL is still unclear. Likewise, the function of these cells has already been described for human cutaneous leishmaniasis during the active and healing phases [44–47]. Some studies have showed that CD8^+^ T lymphocyte cytotoxic activity, secretion of cytokines and chemokines, granzyme B levels and lymphoproliferation may contribute to the parasite control in experimental and human VL [48–51]. At the same time, there was a positive correlation between the CD8^+^ T lymphocytes and transaminases levels, which raises the hypothesis that liver damage could be associated with cytotoxic activity. Herein, this occurred regardless of the VL clinical outcome. Such an association has already been demonstrated in hepatitis B [52] and in infectious mononucleosis by Epstein-Barr virus [53].

Also, endogenous IL-10 secretion has already been ascribed to the CD8^+^ T cells in VL which may contribute to the immunosuppression. Simultaneously, the chronic immune activation may lead CD8^+^ T lymphocytes to express molecules with inhibitory function on their surface, such as PD-1, CTLA-4 [54, 55], TIM-3 and LAG3 [16]. In these situations of exhaustion of the cellular response, CD8^+^ T lymphocytes function may be severely impaired in active phase of VL [56].

In parallel, the levels of specific anti-*Leishmania* IgG and their subclasses after active VL may have an important role as marker of cure and/or predisposition to relapse [17, 57]. Here, IgG3 levels were persistently increased in R-VL group in relation to NR-VL, which may indicate  the presence of continuous parasite stimulation, without a complete control by the immune system. *L. donovani* Indian relapsing patients [58, 59] have shown similar behavior for IgG1, whose levels remained high after the active episode of VL. In other studies [60–62], a reduction in IgG1 and IgG3 levels was found in patients infected either by *L*. *infantum* or by *L. donovani*, who were considered clinically cured few months after the treatment. In addition, we have found that a negative association between IgG3 and CD4^+^ T-cell count in R-VL patients, corroborating the link between persistence of high levels of IgG3 and relapse.

Finally, we observed that IL-6, a cytokine known to be inflammatory and already associated with a more severe prognosis in the evolution of VL [12, 17], was correlated to laboratory severity markers, such as hypoalbuminemia, leukopenia and thrombocytopenia. As expected, CRP levels also correlated positively with IL-6 since its binding with its receptor activates immunocompetent and hematological cells leading to the production of acute phase responses, as CRP [63]. However, there were patients that despite presenting clinical signs and laboratorial data associated with severity did not present elevated IL-6 levels, showing that other mechanisms in VL can trigger immunopathological mechanisms related to severity. This study design and the analysis performed until now are not sufficient to state the role of IL-6 may in the VL relapsing course. This study has limitations, including the small sample, the short follow-up time and mainly the comparison between patients enrolled at the primary but also at a relapsing VL episode, which can add differences in the total time of infection and in previous exposure to anti-*Leishmania* treatments. However, our results are hypothesis generator and rise important questions to be evaluated in a prospective study, as the potential of CD4^+^ T-cell count or IgG3 as biomarkers for VL relapses.

## Conclusion

Therefore, the VL relapsing course among patients not infected with HIV and without other recognized associated immunosuppression is a challenging reality considering the lack of understanding of the pathophysiological mechanisms involved, of reliable prognostic markers and clinical protocols for addressing the condition. Our findings suggest a deficit in T-cell reconstitution and maintenance of B-cell compartment activation as possible immunomechanisms underlying the VL relapse.

## Supplementary Information

**Additional file 1: Supplementary Table 1.** Laboratorial characteristics of non-relapsing and relapsing VL patients during the active phase.

**Additional file 2.** Flow diagram of the study.

## Data Availability

All data generated and/or analysed during this study are included in this published article [and its supplementary information files]. The datasets generated and/or analysed during the current study are not publicly available due individual privacy of patients could be compromised but are available from the corresponding author on reasonable request.

## Abbreviations

6mpt: Six months post-treatment
12mpt: Twelve months after treatment
ADHD: Attention deficit hyperactivity disorder
ALT: Alanine aminotransferase
Amph. B deoxychol: Amphotericin B deoxycholate
Amph. B Lipid Complex: Amphotericin B lipid complex
AST: Aspartate aminotransferase
CRP: C-reactive protein
DB: Direct bilirubin
EI: Elisa Index
F: Female
HS: Healthy subjects
IQR: Interquartile range
Liposomal Amph. B: Liposomal amphotericin B
LPS: Lipopolysaccharide
M: Male
NA: Not applicable
NR-VL: Non-relapsing patients
OD: Optical density
Post-treatment: Right after treatment
R-VL: Relapsing patients
sCD14: Soluble CD14
sCD163: Soluble CD163
Scr: Serum creatinine
VL: Visceral leishmaniasis
yo: Years old
