# Supplementary material for: High levels of anti-Leishmania IgG3 and low CD4+ T cells count were associated with relapses in visceral leishmaniasis
Source: BMC Infect Dis. 2021 Apr 20;21:369. doi: 10.1186/s12879-021-06051-5 (PMC8056614; doi:10.1186/s12879-021-06051-5)
Supplement: Supplementary file 1 — Additional file 1: Supplementary Table 1. Laboratorial characteristics of non-relapsing and relapsing VL patients during the active phase. [file 12879_2021_6051_MOESM1_ESM.doc]

**High levels of anti-*Leishmania* IgG3 and low CD4^+^ T cells count were associated with relapses in visceral leishmaniasis**

**Renata Caetano Kuschnir^1^; Leonardo Soares Pereira^2^; Maria Rita Teixeira Dutra^2^; Ludmila de Paula^2^; Maria Luciana Silva-Freitas^1^; Gabriela Corrêa-Castro^1,3^; Simone da Costa Cruz Silva^4^; Glaucia Cota^5^; Joanna Reis Santos-Oliveira^1,3^; Alda Maria Da-Cruz^1,6,7#^**

*^1^ Laboratório Interdisciplinar de Pesquisas Médicas - Instituto Oswaldo Cruz/FIOCRUZ, Rio de Janeiro, Brazil.*

*^2^ Hospital Eduardo de Menezes, Fundação Hospitalar do Estado de Minas Gerais, Minas Gerais, Brazil*

*^3^ Núcleo de Ciências Biomédicas Aplicadas, Instituto Federal de Educação, Ciência e Tecnologia - IFRJ, Rio de Janeiro, Brazil.*

*^4^Instituto Nacional de Infectologia Evandro Chagas – Fiocruz, Rio de Janeiro, Brazil.*

*^5^ Instituto René Rachou – FIOCRUZ, Minas Gerais, Brazil.*

*^6^ Disciplina de Parasitologia, DMIP, Faculdade de Ciências Médicas, UERJ, Rio de Janeiro, Brazil.*

*^7^ Rede de Pesquisas em Saúde do Estado do Rio de Janeiro/ FAPERJ, Rio de Janeiro, Brazil.*

**# Corresponding author:**

Dr. Alda Maria Da-Cruz, M.D.

Laboratório Interdisciplinar de Pesquisas Médicas, Instituto Oswaldo Cruz, FIOCRUZ, Av. Brasil 4365, Manguinhos, Rio de Janeiro-RJ, Brazil. 21040-360,

Phone: +55.21.2562-1039

Email: [alda@ioc.fiocruz.br](about:blank)

**Supplementary Table 1. Laboratorial characteristics of non-relapsing and relapsing VL patients during the active phase.**

|  | **Patient** | **Sex** | **Age** | **Hemoglobin**  (g/dL) | **Hematocrit**  (%) | **Leucocytes**  (cell/mm³) | **Eosinophil**  (cell/mm³) | **Basophils**  (cell/mm³) | **Neutrophils**  (cell/mm³) | **Lymphocytes**  (cell/mm³) | **Monocytes**  (cell/mm³) | **Platelet**  (x10^3^cell/mm³) | **Urea**  (mg/dL) | **Creatinine**  (mg/dL) | **AST**  (U/L) | **ALT**  (U/L) | **Total bilirubin**  (mg/dL) | **Direct bilirubin**  (mg/dL) | **Indirect bilirubin**  (mg/dL) | **Total protein**  (g/dL) | **Albumin**  (g/dL) | **Globulin**  (g/dL) | **CRP**  (mg/dL) |
| --- | --- | --- | --- | --- | --- | --- | --- | --- | --- | --- | --- | --- | --- | --- | --- | --- | --- | --- | --- | --- | --- | --- | --- |
| Reference |  |  |  | 13.5-17.5 | 41-  53 | 4000-11000 | 0-500 | 0-200 | 1600-7000 | 920-5000 | 80-1000 | 150-450 | F:  15-36  M:  19-43 | F:  0.5-1.0  M:  0.7-1.3 | F:  14-36  M:  17-59 | F:  9-52  M:  21-72 | 0.2-1.3 | 0.0-0.4 | 0.0-1.1 | 6.3-8.2 | 3.5-5.0 | 1.4-3.2 | <10 |
| **Non-Relapsing** | **VL01** | M | 27 | 10.5 | 30.9 | 1700 | 0 | 0 | 765 | 816 | 119 | 92 | 31.2 | 0.9 | 295 | 187 | 0.77 | 0.32 | 0.44 | 9 | 2.5 | 6.5 | 31 |
|  | **VL02** | M | 18 | 9.3 | 29 | 3300 | 0 | 0 | 1419 | 1683 | 198 | 73 | 32.8 | 0.8 | 294 | 137 | 0.3 | 0.3 | 0 | 7.1 | 2.7 | 4.4 | 46 |
|  | **VL03** | M | 48 | 6.9 | 20.4 | 1300 | 13 | 0 | 637 | 546 | 104 | 77 | 49.4 | 1.2 | 50 | 49 | 0.9 | 0.7 | 0.2 | 6.8 | 2.6 | 4.2 | 56 |
|  | **VL04** | M | 32 | 9.6 | 28.8 | 2000 | 40 | 0 | 1040 | 780 | 160 | 51 | 33.4 | 0.7 | 70 | 67 | 0.9 | 0.8 | 0.1 | 5.8 | 2.3 | - | 130 |
|  | **VL05** | M | 44 | 6.1 | 19.9 | 1000 | 0 | 0 | 540 | 360 | 100 | 134 | 23.1 | 0.9 | 59 | 34 | 0.6 | 0.4 | 0.2 | 7.8 | 2.4 | 5.4 | 62 |
|  | **VL08** | M | 45 | 11.1 | 34 | 1300 | 0 | 0 | 559 | 663 | 78 | 18 | 29.6 | 0.6 | 118 | 57 | 0.7 | 0.5 | 0.2 | 6.7 | - | - | 262 |
|  | **VL11** | M | 61 | 7.1 | 22.8 | 900 | 27 | 9 | 252 | 549 | 63 | 11 | 35.3 | 1.2 | 69 | 30 | 0.9 | 0.7 | 0.2 | 8.1 | 2 | 6.1 | 86 |
|  | **VL12** | F | 32 | 6 | 18.2 | 1100 | 0 | 0 | 495 | 495 | 110 | 66 | 31.8 | 0.3 | 44 | 26 | 1.2 | 0.8 | 0.4 | 7.4 | 1.9 | 5.5 | 228 |
|  | **VL13** | F | 32 | 6.6 | 21.1 | 1400 | 14 | 0 | 770 | 490 | 126 | 70 | 14.5 | 0.5 | 163 | 71 | 0.9 | 0.6 | 0.3 | 6.7 | 3 | 3.7 | 82 |
|  | **VL14** | M | 46 | 4.7 | 14.1 | 900 | 27 | 9 | 162 | 540 | 162 | 60 | 41 | 1.3 | 68 | 18 | 1.3 | 1.1 | 0.2 | 9.3 | 2.5 | 6.8 | 31 |
|  | **Median**  **(IQR)** | NA | **38**  (30.8-46.5) | **7**  (6.1-9.8) | **21.95**  (19.5-29.5) | **1300**  (975-1775) | **6.5**  (0-27) | **0**  (0-2.3) | **598**  (434.3-837.5) | **547.5**  (493.8-789) | **114.5**  (94.5-160.5) | **68**  (42.8-80.8) | **32.3**  (28-36.7) | **0.85**  (0.6-1.2) | **69.5**  (56.8-195.8) | **53**  (29-87.5) | **0.9**  (0.7-1.0) | **0.65**  (0.4-0.8) | **0.2**  (0.2-0.3) | **7.25**  (6.7-8.3) | **2.5**  (2.2-2.7) | **5.5**  (4.3-6.4) | **72**  (42.3-154.5) |
| **Relapsing** | **VL06** | M | 37 | 9.1 | 28.2 | 2200 | 44 | 0 | 858 | 1100 | 198 | 119 | 28 | 1.1 | 20 | 22 | 0.5 | 0.3 | 0.2 | 9.2 | 3.2 | 6 | 47 |
|  | **VL07** | M | 23 | 9.5 | 29.5 | 1700 | 17 | 0 | 918 | 578 | 187 | 107 | 25.5 | 0.9 | 28 | 29 | 0.8 | 0.3 | 0.5 | 7.2 | 3 | 4.2 | 212 |
|  | **VL09** | M | 61 | 7.3 | 23.8 | 700 | 0 | 0 | 294 | 273 | 133 | 94 | 57.3 | 1.8 | 38 | 22 | 0.5 | 0.2 | 0.3 | 8.9 | 3.1 | 5.8 | 37 |
|  | **VL10** | M | 62 | 8 | 25.1 | 1500 | 15 | 0 | 600 | 750 | 135 | 82 | 15.6 | 0.5 | 101 | 82 | 0.7 | 0.5 | 0.2 | - | - | - | 62 |
|  | **VL15** | F | 44 | 6.3 | 20.6 | 2200 | 22 | 0 | 1364 | 748 | 66 | 314 | 22.9 | 0.4 | 57 | 36 | 0.4 | 0.4 | 0 | - | 2.4 | - | - |
|  | **Median**  **(IQR)** | NA | **44**  (30-61) | **8**  (6.8-9.3) | **25.1**  (22.3-28.9) | **1700**  (1100-2200) | **17**  (7.5-33) | **0**  (0-0) | **858**  (447-1141) | **748**  (425.5-925) | **135**  (99.5-192.5) | **107**  (88-216.5) | **25.5**  (19.3-42.7) | **0.9**  (0.5-1.5) | **38**  (24-79) | **29**  (22-59) | **0.5**  (0.5-0.8) | **0.3**  (0.3-0.5) | **0.2**  (0.1-0.4) | **8.9**  (7.2-9.2) | **3.05**  (2.6-3.2) | **5.8**  (4.2-6) | **54.5**  (39.5-174.5) |
|  | ***p* value** | NA | 0.49 | 0.76 | 0.59 | 0.39 | 0.31 | 0.52 | 0.44 | 0.67 | 0.32 | **0.01** | 0.29 | 0.98 | **0.03** | 0.30 | **0.03** | **0.06** | 0.77 | 0.27 | **0.06** | 0.96 | 0.76 |

**ALT:** alanine aminotransferase; **AST:** aspartate aminotransferase; **F:** female; **IQR**: interquartile range; **M:** male; **NA:** not applicable; **CRP:**  C-reactive protein; **VL:** visceral leishmaniasis. *p* value: significant difference between non-relapsing and relapsing VL patients.
