# Supplementary material for: High levels of anti-Leishmania IgG3 and low CD4+ T cells count were associated with relapses in visceral leishmaniasis
Source: BMC Infect Dis. 2021 Apr 20;21:369. doi: 10.1186/s12879-021-06051-5 (PMC8056614; doi:10.1186/s12879-021-06051-5)
Supplement: Supplementary file 2 — Additional file 2. Flow diagram of the study. [file 12879_2021_6051_MOESM2_ESM.doc]

Flow diagram of the study.

**Cure**

Absence of fever; recovery of cytopenia and splenomegaly involution (if present)

**Relapses**

Resurgence of fever, worsening cytopenia or increased splenomegaly 🡪 direct examination or culture in NNN medium

**Inclusion:** Fever, cytopenia or splenomegaly, >18 years, independently of gender

**Exclusion:**

Pregnants and

HIV-Co-infection

**NR-VL patients (n=10)**

**Non-relapsing**

experienced only one VL episode throughout life

**R-VL patients**

**(n=5)**

**Relapsing**

experienced more than one VL episode (previous and/or during the prospective follow-up)

**Healthy subjects (n=10)**

**Active phase**

(before treatment)

**Post-treatment**

(immediatly after the final treatment)

**6 months post-treatment (mpt)**

**12 months post-treatment (mpt)**

**Clinical signs and symptoms**

Fever, asthenia, hyporexia, weight loss, vomiting, diarrhea, bleeding, pallor, edema, splenomegaly, hepatomegaly, jaundice and underlying bacterial infection

**Laboratory data**

Blood cell counts, biochemical tests (liver and kidney function)

**Immunological data**

CD4^+^/CD8^+^ T lymphocytes count (peripheral blood); anti-*Leishmania* IgG, IgG1 and IgG3 immunoglobulins and IL-6 levels (plasma)

**NR-VL (n=10)**

**R-VL (n=5)**

**NR-VL (n=9)**

**R-VL (n=5)**

**NR-VL (n=5)**

**R-VL (n=4)**

**NR-VL (n=5)**

**R-VL (n=3)**

**VL patients**

**(n=15)**

HEM-FHEMIG

Belo Horizonte, MG, Brazil

**Diagnosis**

Parasitological: direct research of amastigotes in bone marrow aspirate; Serologic: anti-*Leishmania* antibodies in serum

**Treatment**

Amph. B deoxycholate

Liposomal Amph. B

Amph. B Lipid Complex
